# Budgeting based on need: a model to determine sub-national allocation of resources for health services in Indonesia

**DOI:** 10.1186/1478-7547-10-11

**Published:** 2012-08-29

**Authors:** Tim Ensor, Hafidz Firdaus, David Dunlop, Alex Manu, Ali Ghufron Mukti, Diah ayu Puspandari, Franz von Roenne, Stephanus Indradjaya, Untung Suseno, Patrick Vaughan

**Affiliations:** 1 Nuffield Centre for International Health and Development, Leeds Institute for Health Sciences, University of Leeds, Leeds, LS29LJ, United Kingdom; 2 Pusat-KPMAK (Center for Health Financing Policy and Insurance Management), Faculty of medicine, Universitas Gadjah Mada, Gedung Radioputro, Lt 2 Sayap Barat.Jl. Farmako, Sekip Utara, Yogyakarta, 55281, Indonesia; 3 Consultant to the Center of Health Economics and Policy Analysis and Lecturer, School of Public Health, University of Indonesia, Depok, 16424, Indonesia; 4 Associate Consultant, Oxford Policy Management, 6 St Aldates Courtyard, 38 St Aldates, Oxford, OX1 1BN, United Kingdom; 5 Department of Public Health, Faculty of Medicine, Universitas Gadjah Mada, Jl Farmako, Sekip Utara, Yogyakarta, 55281, Indonesia; 6 Diah Ayu Puspandari, Center for Health Financing Policy and Insurance Management (Pusat KP-MAK), Fakultas Kedokteran, Universitas Gadjah Mada, Gedung Radyoputro, Lt 2 Sayap Barat, Jl. Farmako, Sekip Utara, Yogyakarta, 55281, Indonesia; 7 Head of the Health Section, Abteilung Bildung, Gesundheit, Soziale Sicherung, Division of Education, Health, Social Protection, Deutsche Gesellschaft für Internationale Zusammenarbeit (GIZ) GmbH, Postfach 5180, Eschborn, 65726, Germany; 8 GIZ Consultant, Pluit Kencana V/9, Jakarta, 14450, Indonesia; 9 Senior advisor to the Minister of Health Republic of Indonesia, Jl HR Rasuna Said blok x-5 kav 4-9, Jakarta, 12950, Indonesia; 10 Professor Emeritus in Epidemiology and Public Health at the London School of Hygiene and Tropical Medicine, and Associate Consultant to Oxford Policy Management, London, United Kingdom

**Keywords:** Resource allocation, Costing, Benefits package

## Abstract

**Background:**

Allocating national resources to regions based on need is a key policy issue in most health systems. Many systems utilise proxy measures of need as the basis for allocation formulae. Increasingly these are underpinned by complex statistical methods to separate need from supplier induced utilisation. Assessment of need is then used to allocate existing global budgets to geographic areas. Many low and middle income countries are beginning to use formula methods for funding however these attempts are often hampered by a lack of information on utilisation, relative needs and whether the budgets allocated bear any relationship to cost. An alternative is to develop bottom-up estimates of the cost of providing for local need. This method is viable where public funding is focused on a relatively small number of targeted services. We describe a bottom-up approach to developing a formula for the allocation of resources. The method is illustrated in the context of the state minimum service package mandated to be provided by the Indonesian public health system.

**Methods:**

A standardised costing methodology was developed that is sensitive to the main expected drivers of local cost variation including demographic structure, epidemiology and location. Essential package costing is often undertaken at a country level. It is less usual to utilise the methods across different parts of a country in a way that takes account of variation in population needs and location. Costing was based on best clinical practice in Indonesia and province specific data on distribution and costs of facilities. The resulting model was used to estimate essential package costs in a representative district in each province of the country.

**Findings:**

Substantial differences in the costs of providing basic services ranging from USD 15 in urban Yogyakarta to USD 48 in sparsely populated North Maluku. These costs are driven largely by the structure of the population, particularly numbers of births, infants and children and also key diseases with high cost/prevalence and variation, most notably the level of malnutrition. The approach to resource allocation was implemented using existing data sources and permitted the rapid construction of a needs based formula that is highly specific to the package mandated across the country. Refinement could focus more on resources required to finance demand side costs and expansion of the service package to include priority non-communicable services.

## Background

Population health service needs are regionally specific in most countries. National health systems are faced with the dilemma of allocating resources to these areas to take account of need. Although historic, incremental systems were the norm in the past, countries increasingly make use of information on local needs to influence these allocations.

The main approach to resource allocation has been to identify variables that explain need within a community and use them to develop weights for allocating resources between areas [1]. Early formulae included relatively crude proxies for need such as the (under 75) Standardised Mortality Rate (SMR) which was used in the UK as a proxy for morbidity from the early 1970s [2]. This has given way to a more complex understanding of need that examines the relationship between key need indicators such as age, sex and reported health status and measures of morbidity as expressed through current utilisation adjusted for the availability of services. As well as direct measures of need, formulae also draw upon indirect, distal measures of need such as socio-economic status such as unemployment, elderly people living alone and children of single parents [1].

Formulae depend on the accuracy of proxies and weights used which can often appear arbitrary. As shown by one study in Canada, the final allocations can be very sensitive to the choice of variables used to proxy need [3]. The desire for empirically verifiable proxies and weights has led to the use of econometric models that examine the association between use of services and a wide range of need variables. The resulting coefficients can be used as weights in an adjusted allocation formula. Historically, most systems have used actual utilisation data of specific groups as a measure of future resource need. This approach has the advantage that data is relatively easy to obtain but may fail to account for the resource requirements of those that have need but do not currently utilise services (unmet need) [4]. Early studies were criticised because they failed to take account of the effect of supplier induced utilisation . Later studies attempted to control for the supply effect through more sophisticated modelling [5,6]. Area based formula funding is now used by a large number of OECD countries in Western Europe, Canada and Australasia [1].

Formula funding for health is beginning to be used in low and middle income countries South Africa, Zambia and Namibia, for example, all allocate resources for health based on population adjusted for a variety of need factors [7]. In Bangladesh studies have examined how different weights on poverty and health outcomes would impact on resource distribution [8]. These approaches have the important feature that they de-link the allocation of resources from historic levels of supply and so have the potential to produce important shifts of resources towards areas with low use relative to need. Statistical analysis of need factors to determine weightings is hampered by the lack of large scale data sets representative at area level in most low and middle income countries. Demographic and Health Surveys (see measuredhs.com) provide what are possibly the most consistent and reliable datasets for the modelling of health needs but are usually not representative below the level of province and are largely restricted to a set of mainly process indicators for maternal and child health utilisation. Weightings may also be politically influenced. One study in Ghana, for example, found that the weightings chosen for the formula allocation of local government grants were ‘amended to produce politically desired patterns of transfers’ [9].

A second stage of the resource allocation process is a decision on the level of resource to allocate for the measured needs of each area and population sub-group. In well established health systems the usual method is to assume the unit of resource is the available total budget and allocate it based on the need weighting. Where there is no established budget an alternative is to build up resource requirements by looking at individual services based on epidemiological data and the normative cost of treatment [10]. This approach was used to analyse need for services for angina and myocardial infarction using patient data in Wales [11]. The complexity of universal needs packages in most OECD countries limits the extent to which this methodology can be applied to most services. In contrast, a low level of resources and intention to direct them to priority needs mean that many Low and Middle Income Countries (LMICs) aim to focus public funding for health care on a limited range of interventions that are of proven cost-effectiveness. A basic benefit package approach, focusing on a narrow range of mostly communicable disease and maternal and child health, has become a common feature of country sector strategies in many LMICs. The approach has been central to international initiatives advocating more but better targeted spending on health care [12,13]. A bottom up, approach to need for resource allocation may be practical for the limited range of services financed by the state in such countries and be more specific to needs than a general formula.

We describe a bottom-up approach to developing a formula for the allocation of resources in Indonesia. The data requirements are no greater than those for a more general proxy formula. The approach is general but was developed for Indonesian regions as part of a larger costing study. The first section describes the current decentralised context for the funding of health care in Indonesia. Section two, describes the methods used to establish the normative costs of the package and the production of context specific scenarios. Results for a number of actual and average contexts are then presented to illustrate their variability and main determinants. Finally, the findings are discussed in the context of health policy in relation to geographic resource allocation in Indonesia and applications internationally.

### Decentralised funding of health care in Indonesia

In Indonesia, the inequity of historic funding patterns is exacerbated by the effects of the decentralisation law enacted in 1999 and revised in 2001 which placed most health services under the responsibility of district government [14]. District Health Offices must now compete with other sectors for funding. A recent public expenditure review suggested that local health spending is largely associated with revenues of the district rather than population need [15]. Inequity at the district level is likely to contribute to inequity at the individual level: research studies indicate that Indonesia has one of the least pro poor distributions of public health resources in the region [16].

The Ministry of Health maintains influence over the health system by mandating local authorities to provide a minimum package of services (Standard Pelayanan Minimal or SPM) to their population. The SPM comprises maternal and neonatal care, family planning, infant and child health (including routine health checks and care for children suffering from malnutrition, diarrhoea and respiratory infections) and priority communicable diseases (tuberculosis, malaria and dengue). The SPM establishes target levels of coverage of relevant population groups ranging from around 75% (coverage of malaria cases) to 95% (antenatal care). The package includes both personal health care and public health measures (e.g. spraying for dengue). The package also defines support for a wider range of personal health care for the poor including basic and referral care. These services are not well defined and in this paper we confine attention to the cost of the universal services that are specified to be provided to the entire population. .

Financing to achieve SPM targets comes from several sources. Since 2004 there has been an insurance scheme for the poor based on household and individual characteristics first administered by the state health insurance agency (PT Askes) and, since 2008, as a special programme of the Ministry of Health (Jamkesmas). Districts are required to make funding available for the priority services to the remainder of the population but little is known about the costs of such commitments and how they vary across the country. Wide variation in costs is probable since the country is made up of more than 17,000 islands which have widely different economic and social status (Table 1). The focus of the study is on the estimation of the funding required in order to achieve the minimum SPM coverage level defined politically for each service across different regions of the country taking into account variations in demography and epidemiology. Sources: [17]; [18]

**Table 1:** Selected regional descriptive statistics for Indonesia

	**Severe Malnutrition (%)**	**No education (females) (%)**	**Total fertility rate**	**% couples using modern method family planning methods**	**Infant population (%)**	**Population density**	**Infant Mortality Rate (per 1000 infants)**	**Skilled provider delivery**
DKI Jakarta	17.0	3.5	2.1	56.4%	1.3	12,635	28	97.3
Yogyakarta	9.0	15.5	1.8	66.9%	1.1	980	19	95.8
West Nusa Tenggara	15.5	17.4	2.8	55.2%	1.8	199	72	64.3
Central Java	11.8	14.8	2.3	60.0%	1.3	959	26	83.0
West Papua	16.4	33.6	2.9	37.5%	1.2	-	36	57.7
High	22.1	3.9	4.2	74.0%	2.3	12,635	74	97.3
Low	9.0	33.6	1.8	34.1%	1.1	6	19	32.8
INDONESIA	13.6	11.7	2.6	61.4%	1.5	109	34	73.0

## Methods

The bottom-up allocation approach focuses on the cost of providing a package of essential services in a typical district in each of the 33 provinces of Indonesia. To investigate the impact of different district contexts on the costs of SPM, the condition specific sheets were linked to demographic (age and sex), epidemiological (proportion of the population suffering each disease) and location data (distance between health centres and hospitals for referral). A user friendly interface to enter data and undertake simulations was constructed based on user forms in Visual Basic.

The modelling incorporated a representation of a typical district structure. In Indonesia, public services are usually organised around one (sometimes more) district hospitals and sub-district based health centres (Puskesmas). Health centres are of two main types: those with beds primarily for emergency obstetric care, and those without beds. The health centre acts as a focal point for other public services in the sub-district including sub-health centres, village health posts and services of village midwives. The latter have been an important part of the government’s strategy to expand use of skilled delivery care since the early 1990s [19]. These services are incorporated as part of the Puskesmas network for costing.

The total allocation for *s* SPM services in district/province *i* is given as

(1) $$Total\;Cost\;{\left(SPM\right)}_{i}=\overset{S}{\underset{j=1}{\sum }}t_{\mathrm{ij}}d_{\mathrm{ij}}n_{j}c_{\mathrm{ij}}$$

Where *t*_*ij*_ is the proportion of the total population (target group) that potentially may require the service j, *d*_*ij*_ is the proportion of the target group that are expected to present with the condition and *n*_*j*_ is the proportion with the disease that require treatment at a medical facility. The latter reflects the fact that for some conditions treatment is not required or can be provided at home without recourse to medical facilities. Unit costs (c) for service j are assumed to vary across districts and provinces (i):

(2) $$c_{\mathrm{ij}}=c_{\mathrm{ij}}^{p}+\pi_{j}\left[\left(1-m_{j}\right)c_{\mathrm{ij}}^{H}+m_{j}c_{\mathrm{ij}}^{\mathrm{HA}}+r_{i}\right]$$

Where *c*_*i*_*j*^*p*^ , *c*_*ij*_^*pH*^ and *c*_*ij*_^*HA*^ are respectively the unit cost of primary, hospital care without admission and hospital care with admission for service j in district i; *π*_*i*_ is the proportion requiring referral for hospital treatment, *m*_*i*_ is the proportion of referrals requiring admission and *r*_*i*_ is the average cost of referral from primary to hospital facilities in district i. The cost of care varies by region because of differences in fixed costs, variations in the health system and differences in whether health centres provide inpatient care. The unit cost of drugs, medical supplies and direct staffing are established normatively and are assumed not to vary across the country. Base information for the proportion of target groups with particular conditions was taken from the Department of Health’s regular survey and the most recent Demographic and Health Survey [17,18].

The cost of primary and hospital care are composed of variable (v) elements such as drugs, laboratory tests and supplies; direct staffing (s) required in the delivery of the specific service by midwives, nurses and midwives; and fixed overhead costs (f) arising from the operational, capital and indirect staffing (administration, clinical support departments and ancillary staff) of each facility or cluster of facilities.:

(3) $$c_{\mathrm{ij}}^{p}=v_{j}^{p}+s_{j}^{p}+f_{\mathrm{ij}}^{p}$$

(4) $$c_{\mathrm{ij}}^{H}=v_{j}^{H}+s_{j}^{H}+f_{\mathrm{ij}}^{H}$$

Treatment normatives for particular diseases are assumed to be similar so that the variable and direct staffing inputs are the same. The variable (v) and direct staffing (s) elements of the cost of each SPM condition were derived through a process of consultation with programme directors in the Ministry of Health and clinicians working within facilities. For variable items this involved listing the drugs and medical supplies required in the treatment of each condition together with quantities required and the probability of a typical patient with the condition requiring the item. For direct staffing, it involved quantifying the time spent by key health staff (general doctors, specialists such as obstetricians, midwives and nurses) with each patient during an episode of illness. To aid this process staff time was listed by key activity area such as time during admission, in the operating theatre, each day on the ward and at discharge. Costs were entered on series of sheets adapted from the Core Plus costing framework and WHO Mother and Baby Package [20,21]. Costing involved a number of iterations since estimates of staff time were thought to be exaggerated and were revised after preliminary total costs were produced. Resource requirements are based on best local clinical practice for each condition.

Fixed overhead costs are permitted to vary across districts as they are directly related to the number of facilities that are required to serve a given population. Facility numbers are influenced by geography and topology so that a sparsely populated mountainous district will require, ceteris paribus, a larger number of facilities to serve population need. Similarly, while it is assumed that the proportion of patients with disease j requiring referral is similar across districts, the cost of referral (r) is influenced by proximity to referral facilities and so will vary across districts.

The costing incorporates three types of fixed overhead:

1. Facility overheads (health centre or district hospital), including administrative and support staff, operating costs of the facility and (annualised) capital

2. SPM overheads attributable to particular SPM services (e.g. spraying for dengue, surveillance for infectious diseases) which vary on a population basis rather than according to patients treated. These services are largely provided through the DHO and the costs are apportioned to each facility and the service as part of the modelling.

3. Administrative overheads associated with running the DHO

We include all the costs of treatment whether provided at hospital, health centre or in the community. The costing of the health centre (puskesmas) incorporates the costs of subordinated facilities such as sub-centres and village health posts.

The service-based SPMs are largely limited to communicable diseases plus maternal care. Much of the disease burden, for example from non-communicable diseases and trauma, is excluded. Although not directly costed, the model recognises the economies of scope of providing for these other diseases by incorporating an estimate of workload (beds filled, outpatients treated) attributed to other conditions. This activity is then added to the SPM activity in order to allocate overhead.

Distance costs are important for the health system and individual patients, particularly in more remote areas of Indonesia where distances (measured in time or distance) between populations and facilities are large (due to oceans or mountains). The costing focuses on system costs and does not incorporate the costs to households of getting to a facility for initial treatment. These demand-side costs are important in explaining the difference between normative need and actual demand [22]. The costing assumes that the system is responsible for patient transport between health centre and hospital where referral is medically necessary. The model incorporates a simple map that computes the straight line distance between all health centres and the district hospital(s). These distances are used to impute an emergency transport cost for patient transfers. This is clearly a simplification since transport links are not usually straight line. Furthermore some terrain may be more difficult (costly and slower) to travel on than others. This is particularly true where transport to a hospital necessitates crossing water. The costs derived, therefore, should be regarded as a minimum - local knowledge is required to augment the estimates. An extension of the model could incorporate more accurate time mapping based on GIS data.

## Results

The model was initially estimated from data from local district staff describing the demographic structure and scale mapping of sub-districts and local facilities for five districts: Yogyakarta (DI Yogyakarta province), Purbalinga (Central Java province), Lombok Barat, Dompu and Lombok Tengah (West Nusa Tenggara province). Initial estimates helped identify key drivers of cost affecting inter-district differences in per capita cost. The model was then re-estimated for a typical district in each province.

The most expensive individual SPM conditions were found to be treatment both for delivery complications and neonatal (intensive) care (Table 2). The cost per episode of malnutrition was also high. Rates of malnutrition are high in many areas of Indonesia and require a period of intensive therapeutic feeding in hospital for the most severe cases followed by supplementary feeding in the community (outpatient based). Dengue and permanent family planning methods are expensive per episode. Across the country, the proportion of the population requiring these services is low and so the per capita cost is modest. In contrast, non-permanent methods of contraception are very cheap at an individual level but the per capita cost is relatively high due to large numbers requiring the service.

**Table 2:** Cost per episode of each SPM Service (average cost across sample districts)

**SPM Service**	**Direct supplies in USD per Episode**	**Direct staff in USD per Episode**	**Overhead in USD per Episode**	**Total in USD per Episode**	**% overhead in total**	**Per capital (USD)**
**Maternal Health**						
Basic Antenatal Care	6	9	6	20	29	0.42
Abortion	17	16	57	90	63	0.03
Antepartum Haemorrhage	78	24	190	292	65	0.08
Hypertension PET	590	26	152	238	64	0.04
Severe Anaemia	60	13	115	188	61	0.18
Premature Labour	138	13	67	218	31	0.12
Abnormal foetal presentation	36	25	69	130	53	0.07
Prolonged Labour	30	25	58	113	51	0.11
Caesarean Section	33	47	129	209	62	0.31
Uterine Rupture & Hysterectomy	61	83	180	323	56	0.11
Intrapartum & post partum infection	103	21	165	289	57	0.07
Post Partum Haemorrhage	31	61	129	221	59	0.12
Normal Delivery	12	11	17	41	42	0.46
Routine Post Partum Care	1	15	5	21	23	0.35
**Child Health**						
Neonatal Complications	223	39	137	400	34	0.80
Routine Infant Health	0	7	5	11	41	0.17
Routine Child Health	0	6	9	15	62	0.96
Child Immunisation	17	3	6	26	23	0.29
Nutrition for the Poor	28	4	3	36	9	0.20
Severe Malnutrition	51	23	125	199	63	2.12
School Health	0	2	2	4	42	0.06
**Other Reproductive Health**						
Family Planning Non Permanent	9	2	1	12	12	1.33
Family Planning Permanent	86	17	32	135	24	0.17
**Selected Communicable Diseases**						
Pneumonia	23	8	20	51	38	0.32
Diarrhoea < 5 years	12	8	7	27	25	0.27
Diarrhoea > 5 years	15	8	4	26	15	0.31
Malaria < 5 years	6	10	12	27	44	0.12
Malaria > 5 years	10	10	11	31	36	1.16
Tuberculosis <5 years	45	6	18	70	26	0.05
Tuberculosis >5 years	42	6	19	67	28	0.39
Dengue < 5 years	91	10	37	139	27	0.10
Dengue > 5 years	95	10	34	138	24	0.82

Note: Data provided by each district DHO, or via national statistics where available on a district basis.

Overheads are driven by a variety of factors but a substantial determinant is the extent of hospitalisation required for conditions. The proportion of overhead in total episode costs varies from 11 to 76% across the SPM services. So, the relatively high overhead (almost 60%) for many obstetric conditions arises from the costs of surgery and subsequent recovery in hospital. In contrast, much of the cost of providing immunisation, where direct costs account for 89%, are the costs of vaccines and staff administering the vaccination with little use of facilities. The analysis suggests that a standard overhead, often used in other costing studies, may be unrealistic since the size of the overhead crucially depends on the service mix.

**Figure 1:**
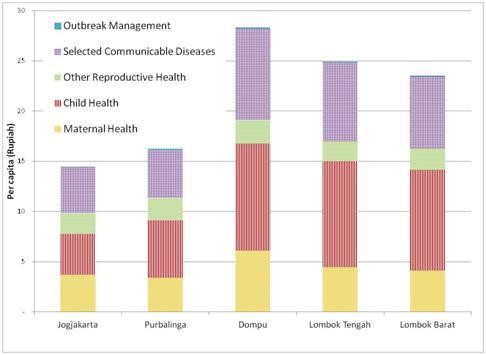
Comparison of per capita costs of SPM services by area and main condition.

The results across the five districts show considerable differences in per capita costs of SPM services (figure 1). These range from less than $15 in Yogyakarta to almost $30 in Dompu.

Differences in costs between categories of service are driven by two factors: a) substantial service cost or b)large case numbers are. Although there are some differences in communicable diseases (dengue, malaria, TB) across the districts the relatively small population affected means that these do not contribute substantially to the difference in costs. In contrast the number of infants and newborns drives costs through the wide range of sometimes expensive conditions (e.g. emergency obstetric and neonatal intensive care) impacting a large population.

The impact of each variable can be demonstrated by computing an elasticity based on the simulations across 28 provinces. This indicates how much the per capita costs change for each percentage increase or decrease in the driver variable (holding other variables constant) (Table 3). The importance of changes in the birth rate is evident as is the proportion of infants under one and children 1 to 5. The impact on per capita costs of the prevalence of communicable diseases is small since the proportion affected with these diseases is small relative to the population.

**Table 3:** Estimates of incremental cost variation and elasticities

		**Provincial variation**			
		**Minimum**	**Average**	**Maximum**	**Elasticity**
**Birth Rate**	Cost (per capita)	$ 21.07	$ 22.82	$ 25.98	11.65
	Level (%)	1.7%	2.5%	3.7%	
**Infants (<1)**	Cost (per capita)	$ 22.29	$ 22.82	$ 23.23	3.51
	Level (%)	1.1%	1.6%	2.3%	
**Children (1–5)**	Cost (per capita)	$ 21.41	$ 22.82	$ 24.59	2.65
	Level (%)	5.2%	8.2%	10.8%	
**Eligible Couples**	Cost (per capita)	$ 22.42	$ 22.82	$ 23.08	0.72
	Level (%)	15.7%	17.3%	19.8%	
**Malnutrition**	Cost (per capita)	$ 22.26	$ 22.82	$ 26.90	1.59
	Level (%)	9.0%	13.0%	22.1%	
**Tuberculosis**	Cost (per capita)	$ 22.54	$ 22.82	$ 23.33	3.44
	Level (%)	0.1%	0.4%	1.1%	
**Malaria**	Cost (per capita)	$ 21.89	$ 22.82	$ 33.21	2.00
	Level (%)	0.3%	2.9%	26.1%	
**Dengue**	Cost (per capita)	$ 22.15	$ 22.82	$ 25.49	6.58
	Level (%)	0.2%	0.6%	2.5%	
**Density**	Cost (per capita)	$ 22.23	$ 22.82	$ 25.64	- 0.15
	Level	980.00	109.00	6.10	

Note: estimates from average districts in 28 provinces.

To demonstrate importance of the population structure on the costs of care, the model was run for a district of average size and geography in 28 provinces based on the population structure from the last census and province specific epidemiological and geographic information (full data were only available for 28 out of 33 provinces).

**Figure 2:**
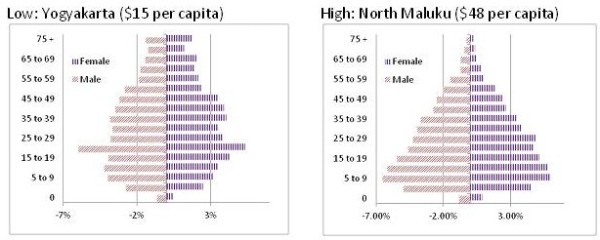
Variations in population structure in lowest and highest SPM cost provinces.

**Figure 3:**
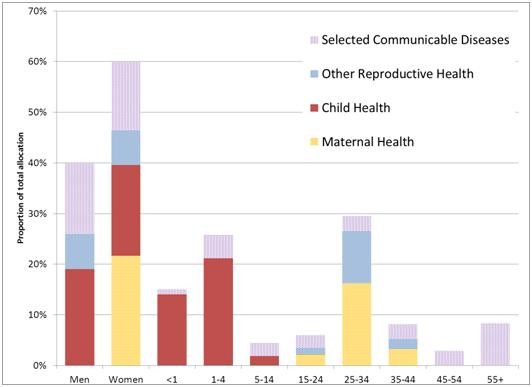
Proportional allocation of spending by age and sex (national average).

The estimates suggest more than a threefold difference between the lowest and highest cost per capita based on need and a four-fold difference when the cost of current (2010) utilisation is assumed (Table 4). The reason for this difference is suggested by the population structure (pyramid) of each province. Yogyakarta, the province with the lowest per capita cost, attracts many students to its universities and is also a popular place to retire (figure 2). As a consequence the province has a disproportionate number of low cost age groups (15–29, 25–29) and a large elderly population. The latter may have high health care needs but these are largely not covered by the SPM. In contrast North Maluku, which has the highest per capita estimate of the SPM, has a population pyramid that is bottom heavy implying high SPM need arising from children, infant and maternal health services (figure 3).

**Table 4:** Estimated per capita costs of meeting SPM needs and current demand (by province)

**Province**	**Per capita cost USD (based on actual utilisation)**	**Per capita cost USD (based on need)**
**TOTAL**	13.13	19.39
North Sumatra	15.00	20.89
West Sumatra	20.32	27.36
Jambi	19.13	25.17
South Sumatera	11.47	18.56
Bengkulu	22.81	27.66
Lampung	11.91	18.00
Bangka Belitung	29.16	36.34
Kepulauan Riau	15.93	21.77
DKI Jakarta	10.12	15.75
West Java	9.63	15.33
Central Java	10.82	16.44
DI Yogyakarta	9.46	15.04
East Java	10.56	16.43
Banten	11.16	17.48
Bali	14.85	19.24
West Nusa Tenggara	13.17	21.44
East Nusa Tenggara	22.80	31.32
West Kalimantan	16.44	25.96
Central Kalimantan	27.52	32.61
South Kalimantan	19.32	25.73
East Kalimantan	24.02	30.02
North Sulawesi	24.36	30.01
Central Sulawesi	20.64	27.03
South Sulawesi	14.25	24.17
Southeast Sulawesi	26.29	32.83
Gorontalo	28.44	36.57
Maluku	25.76	35.79
North Maluku	37.71	48.25
Papua	31.67	37.36

Note: full data were available for 28 out of 35 provinces.

Based on national epidemiology, the bottom-up approach allocates 60% for female conditions and 40% for males. More than 20% of the female allocation is for maternal health and the non-maternal health allocation is pro-male reflecting the slightly higher prevalence of some childhood diseases. Whether these groups actually benefit from funding in this proportion depend on local systems and health seeking practices. Given that the package prioritises services for children and pregnant women it is not surprising that the groups benefiting most from the allocation are the under-fives and women in the 25–34 age group. In contrast the over 55 s, which represent around 11 percent of the population, are expected to benefit from less than 10 percent of the budget allocation for SPM. This will change as the SPM begins to incorporate services aimed at the cure, maintenance and prevention of non-communicable diseases.

## Discussion

### Limitations of the analysis

The modelling undertaken for this analysis has a number of limitations. The modelling excludes services that are provided only for the poor which include non-communicable diseases. This was a pragmatic decision based on the lack of precision about precisely what services are included in the package for the poor.

The burden of disease in Indonesia, particularly in more populous areas, is changing. Non-communicable diseases are becoming more important both in overall utilisation and unit cost terms [23]. In common with most essential packages, the SPM continues to focus on communicable diseases and maternal health which will become less important in terms of the overall disease burden and also the catastrophic impact on households. Development of more complete service packages, and financing from taxation or insurance is becoming more important. The next stage of the normative costing work will be to incorporate the costs of major non-communicable diseases into the costing methodology. A challenge in incorporating these diseases into the approach will be the more complex treatment paths associated with many of these diseases. Early work incorporating common conditions such as a hypertension and diabetes suggests that the approach can be readily applied. More complex and rarer conditions (e.g. kidney failure, breast cancer) with complicated treatment decisions and outcomes present a greater challenge. Further work will be required to examine how the relatively simple condition specific data entry sheets can be modified to accommodate these treatment paths.

Location is currently modelled in a crude way to approximate the differential costs of living in remote compared to densely populated areas. Extensions of the modelling might include more accurate accounting for the costs of distance that, for example, take account of the actual road network and travel times over more difficult terrain.

The cost estimates are based on a normative approach to service provision. While the overhead includes the possibility of variation according to workload, the direct costs are based on nationally determined best practice. Salary costs are based on current levels of pay although we make what we consider to be realistic assumptions about the level of availability of staff during a working day (around 4 hours for doctors and 6 hours for nurses/midwives). The latter assumption takes into account that most staff undertake private practice to supplement their income. A large scale actual cost study is currently underway and it is intended that these normative costs will be compared with the results of the actual costing to test whether these assumptions are correct.

### Policy uses of costing information

The Indonesian government urgently needs to address the resource requirements which the health sector places on each level of government according to the constitution. Current service mandates fail to ensure essential services because the costs required to for delivering on these mandates and the responsibility for providing resources is unclear. Clearer costing that accounts for geographic differences in need provides a basis both for establishing the overall cost of SPMs over the entire country and indicate the level of (considerable) variation in different provinces and districts. Given these costs, policy discussion now needs to focus on the responsibilities for financing services. Local resources will need to be supplemented by national allocations, particularly in areas where fiscal capacity is low. Such accommodation is already implied in the fiscal relationships between levels of government under decentralisation but, in the health sector at least, sound cost estimates have been lacking.

Cost sharing may also include how private financing can be expected to pay for these basic services. Current SPM targets are for between 80 and 95% coverage. Publicly financing to achieve such targets appears to be unaffordable given current public health budgets implying the need for discussion about the total level of public funding and an acceptable split between public and private funding.

Beyond the use of costing for budgeting purposes, the projections of this work highlight the gap between current demand and need. Closing this gap is unlikely to be purely a matter of more funding for services. Whilst the costs of emergency referral between facilities are incorporated into the model, other costs including travel to the first contact provider are borne by consumers. Considerable evidence suggests such demand-side costs are strong, and possibly the dominant deterrent to health seeking behaviour [24]. Funding for strategies to reduce these demand side barriers might be incorporated into resource allocation formulae perhaps by ensuring that some funding is only accessible for mechanisms to reduce these costs such as community transport schemes or funding village health workers to accompany patients for referral.

### Applicability and cost of approach in other contexts

The bottom-up epidemiological approach to costing has been used here to describe how a budget for a limited package of priority services could be allocated across a country. The approach explicitly takes account of area specific epidemiology and demography, proportion requiring treatment and expert assessments of resources required to treat diseases. Proxy data are replaced by information on actual prevalence. The approach requires area specific data down to the level for which the formula is to be used. It is a sensitive way of dealing with need outliers since it incorporates epidemiology weighted by the cost of providing the services in question.

The approach has been demonstrated intensively in Indonesia. Some of the authors have demonstrated the working of a similar approach in Timor Leste and Kenya. The approach does not appear to be any more expensive or time consuming than an approach using proxy variables. In each country, the approach took approximately two months to develop and implement using available secondary data. In Indonesia, demographic and health survey data was supplemented by a large national surveillance survey, Riskesdas, which provides detailed prevalence information across a wide range of communicable and non-communicable diseases including almost all the conditions included in the SPM.

The use of a dynamic model enables the assumptions to be updated with new conditions added to the SPM such as treatment for emerging non-communicable diseases and changing numbers in need.

The modelling undertaken provides estimates of relative costs but also highlights a number of issues related to the way in which the health system is utilised. The overhead elements of condition costs vary considerably and are largely dependent on the need for hospital services for certain conditions. Further development of methodology could focus on whether hospital and primary care services are being used appropriately as the normative treatment for individual conditions. In some cases, such as pregnancy or labour complications, hospitalisation is unavoidable. For conditions such as malnutrition some level of hospitalisation for very young children is required for severe cases but much can (and is) managed at the health centre (puskesmas). Normal deliveries can often be carried out safely at the health centre particularly where the health centre has provision for possible complications or where rapid transport to hospital is possible in an emergency. Further scenario work could examine the impact on cost and quality of referral restructuring and expectations of care at different levels of the system.

## Endnote

^a^In the modelling we additionally make provision for a proportion of the target group with a condition that self-refer for treatment. This is likely to occur even when normatives specifying a gatekeeper role for primary care are rigidly enforced both for emergency treatment for some conditions and in the case of urban populations that rely on hospitals to deliver first-level services.

## Competing interests

The authors declare that they have no competing interests.

## Authors’ contribution

TE wrote the article and developed the model. HF & DD assisted in model development and led the normative costing exercise. AM & PV developed the plan for normative costing and undertook initial interviews. US and SI led the policy discussions about resource allocation .SI & DP collected secondary data. All authors read and approved the final manuscript.
